# A Fuzzy-PI Clock Servo with Window Filter for Compensating Queue-Induced Delay Asymmetry in IEEE 1588 Networks

**DOI:** 10.3390/s24072369

**Published:** 2024-04-08

**Authors:** Yifeng Zhang, Haotian Li, Shixuan Wang, Feifan Chen

**Affiliations:** State Key Laboratory of Precision Measurement Technology and Instrument, Department of Precision Instrument, Tsinghua University, Beijing 100084, China; yifeng-z18@mails.tsinghua.edu.cn (Y.Z.); li-ht21@mails.tsinghua.edu.cn (H.L.); wsx22@mails.tsinghua.edu.cn (S.W.)

**Keywords:** clock synchronization, IEEE 1588 Precision Time Protocol (PTP), queue-induced delay asymmetry, clock servo, packet selection algorithm, fuzzy-PI controller

## Abstract

Clock synchronization is one of the popular research topics in Distributed Measurement and Control Systems (DMCSs). In most industrial fields, such as Smart Grid and Flight Test, the highest requirement for synchronization accuracy is 1 μs. IEEE 1588 Precision Time Protocol-2008 (PTPv2) can theoretically achieve sub-microsecond accuracy, but it relies on the assumption that the forward and backward delays of PTP packets are symmetrical. In practice, PTP packets will experience random queue delays in switches, making the above assumption challenging to satisfy and causing poor synchronization accuracy. Although using switches supporting the Transparent Clock (TC) can improve synchronization accuracy, these dedicated switches are generally expensive. This paper designs a PTP clock servo for compensating Queue-Induced Delay Asymmetry (QIDA), which can be implemented based on ordinary switches. Its main algorithm comprises a minimum window filter with drift compensation and a fuzzy proportional–integral (PI) controller. We construct a low-cost hardware platform (the cost of each node is within USD 10) to test the performance of the clock servo. In a 100 Mbps network with background (BG) traffic of less than 70 Mbps, the maximum absolute time error (max |TE|) does not exceed 0.35 μs, and the convergence time is about half a minute. The accuracy is improved hundreds of times compared with other existing clock servos.

## 1. Introduction

In a Distributed Measurement and Control System (DMCS), there are a large number of nodes driven by independent clocks [1]. Clock synchronization plays a vital role because synchronous measurement, distributed action coordination, etc., require each node to have a common time reference. Due to node size and cost limitations, network-based packet switching [2] is the mainstream clock synchronization method. The network link of the DMCS can be divided into two types: wired and wireless. In the wireless field, especially Wireless Sensor Network (WSN) [3], clock synchronization methods are sensitive to power and complexity. Typical synchronization protocols suitable for WSNs include TPSN [4], RBS [5], FTSP [6], etc. However, in industrial automation, wired communication, including Fieldbus and Industrial Ethernet, still accounts for more than 90% of the global market [7]. In contrast, wireless technology is susceptible to interference and attack and has poor security and reliability, leading to limited applications in the industry.

Typical clock synchronization methods for Industrial Ethernet include Network Time Protocol (NTP) and IEEE 1588 Precision Time Protocol (PTP). NTP [8] completely relies on software synchronization and has strong applicability. It originates from the Internet and can only achieve millisecond accuracy. IEEE 1588-2008 (PTPv2) [9] supports obtaining hardware timestamps, and the accuracy can reach the sub-microsecond level. It is widely used in most fields, such as Smart Grid [10], Seismic Survey [11], Automotive Electronics [12], and Flight Test [13]. White Rabbit (WR) [14], also known as IEEE 1588-2019 [15], uses optical fiber to transmit time and frequency information. Based on PTPv2, it adds Synchronous Ethernet and digital double-mixing phase detection. The accuracy can reach the sub-nanosecond level, effectively meeting the requirements of applications such as particle acceleration control [16] and cosmic ray detection [17]. However, its cost of network construction is high, and the application field is narrow. Since the highest requirement for synchronization accuracy in most industrial applications is 1 μs, PTPv2 is still the most popular version in industrial automation.

The high accuracy of PTP relies on the assumption that the forward and backward delays of packets are symmetrical [18]. Nevertheless, in actual networks, PTP packets will compete with background traffic (BG) packets for transmission and experience random queue delays in network elements such as switches. The higher the network load rate, the more pronounced the Queue-Induced Delay Asymmetry (QIDA). The inconsistency between the assumption and reality leads to time offset estimation error, resulting in synchronization accuracy of tens of microseconds [19]. PTPv2 proposes to use Transparent Clock (TC) [9] to measure the residence time of PTP packets inside the switch. The slave clock compensates for QIDA using this time information, and the accuracy can be improved to the sub-microsecond. However, this dedicated switch supporting the TC is generally expensive (USD 1000–2000 per switch) [20,21], leading to a high cost of large-scale use and poor flexibility.

In addition to QIDA, frequency instability of the crystal oscillator (XO) is also an essential factor affecting PTP accuracy [22]. The XO frequency is susceptible to drift due to the influence of temperature, pressure, and aging. Therefore, time error is easily accumulated, and the clock time must be periodically corrected. Correction methods comprise offset compensation and frequency compensation [23]. Since most nodes are designed based on unidirectional time flow [24], negative time correction can easily lead to system instability, so more scholars adopt frequency compensation. A clock servo is a control system that uses frequency compensation to periodically correct the time offset [25]. There are many studies on clock servo [26,27,28,29,30,31,32,33,34], but few provide a detailed evaluation of synchronization performance under QIDA.

To fill this knowledge gap, this paper designs a PTP clock servo for compensating QIDA. The synchronization accuracy can achieve 1 μs in the network of ordinary switches. Its main algorithm consists of a minimum window filter with drift compensation and a fuzzy-PI controller. The main contributions are as follows:

1. We propose a time offset estimation algorithm based on the minimum window filter. It has two improvements over ref. [35]. One is to use a single observation window to complete the frequency offset estimation without constructing a second window. Another is that the estimation is optimal bidirectional estimation, effectively reducing the estimation error.
2. We propose a method to determine PI coefficients according to the damping ratio and natural frequency under the discrete system model. After adding fuzzy logic, the system can adaptively adjust PI coefficients at different stages, ensuring rapid convergence and high synchronization accuracy. The physical and fuzzy domains of the fuzzy controller are separated by scaling factors, improving the design flexibility.
3. We optimize the algorithm parameters and evaluate the performance of the clock servo based on the broadcast traffic model. In a 100 Mbps network with BG traffic of less than 70 Mbps, the maximum absolute time error does not exceed 0.35 μs, which is improved hundreds of times compared with other clock servos [27,28,31,33].

This paper is organized as follows. Section 2 reviews the research progress of the QIDA compensation methods and design methods of the clock servo. Section 3 analyzes the sources for delay asymmetry and introduces the frequency compensation clock model. Section 4 details the design method of our clock servo. Section 5 presents the experimental platform and BG traffic model. Section 6 discusses the experimental results and compares our method with others. Finally, Section 7 concludes and looks forward to future works. For convenience, a list of symbols and associated definitions adopted in this paper are organized in Table 1.

## 2. Related Works

QIDA and frequency instability of the XO are two main reasons for deterioration in synchronization performance. For QIDA, there are many compensation methods, and scholars were committed to achieving the same performance as PTPv2 in the network of ordinary switches. With regard to the frequency instability of the XO, different clock servos were designed to follow frequency drift. Next, we will detail the research progress.

### 2.1. QIDA Compensation Methods

Currently, QIDA compensation methods (QIDACMs) include PTPv2, probing packets, controlled packet departure, filter, packet selection algorithm, etc. Sending extra probing packets [36] after the regular PTP process can estimate asymmetry. Although it can improve the synchronization performance, it involves some changes to PTP, and the compatibility is not strong. Controlled packet departure [37] allocates a sufficient time slot between PTP packets and BG traffic packets to ensure that the PTP packets will not experience queue delays. This method has strict timing requirements, and its applicability is not strong. Some conventional filters [27,28] are used to mitigate the effect of asymmetry. For example, a low-pass filter can separate high-frequency noise from the frequency domain, and a Kalman filter can achieve optimal performance in estimating Gaussian-distributed noise. However, QIDA noise sometimes contains an impulse component, which is not Gaussian-distributed, and the noise signal frequency also changes with network load. Therefore, conventional filters are not suitable for compensating QIDA, and the accuracy cannot be guaranteed within 1 μs. Compared to conventional filters, the statistical window filter, also known as the packet selection algorithm, performs better in estimating non-Gaussian-distributed noise. Its principle is to obtain enough samples through many packet exchanges and then acquire accurate time offset estimation through statistical operations. Hadzic et al. [38] compared three packet selection strategies (minimum, maximum, and mean). Under the cross-traffic model with a load rate of less than 45%, the sampling minimum performs best in terms of output noise variance. Later, Hadzic et al. [39] proposed an adaptive algorithm that selects the lowest noise variance among the minimum, maximum, and mean at any time as the effectual output. Chaloupka et al. [40] set a large enough window to include the minimum delay packet and verified through simulation that the synchronization accuracy after compensation can reach 1 μs. However, they did not evaluate the algorithm performance on a real hardware platform. Giorgi et al. [41] proposed a new Boltzmann package selection algorithm to verify the feasibility of the frequency transfer using the oversampling strategy. Studies [38,39,41] share a common premise that the time offset remains constant within the window. It is a relatively strict constraint, and the window cannot be selected too long. Freire et al. [35] added preprocessing of drift compensation before packet selection, and the window length is no longer constrained by the constant time offset. Their experimental results show that the minimum or maximum strategy is very effective in compensating for QIDA.

In addition to the packet selection algorithm, there are other compensation methods. For example, Puttnies et al. [42] presented a PTP-linear programming (PTP-LP) method, which uses multiple samples to obtain the upper and lower bounds of the slave clock time and averages the bounds to obtain the estimation value. Nevertheless, the computational cost of solving the LP problem is high. Ha et al. [43] directly modeled delay asymmetry through the linear differential equation and state space model and provided an optimal time offset estimation, and the accuracy can reach 1 μs. However, the estimation algorithm requires real-time measurement of the clock frequency offset using an oscilloscope, making it not easy to implement in practical applications.

The characteristics of the above methods are summarized in Table 2. Furthermore, they have a common problem: they only evaluate the time offset estimation accuracy of the proposed method under asymmetric conditions or directly correct the clock with offset compensation. Few studies combine their methods with frequency compensation.

### 2.2. Design Methods of the Clock Servo

The current mainstream design methods of the clock servo comprise the filter-based proportional–integral (PI) controller, optimal PI controller, and fuzzy-PI controller. PI controller is the control algorithm commonly used in the engineering field, and the integral is used to track the tolerance and unstable jitter of the slave clock XO frequency. A low-pass filter (LF) [26,27] can effectively filter out noises outside the passband but at the cost of introducing phase delay, resulting in deterioration in the dynamic characteristics. Optimal PI [31,32] optimizes the PI coefficients by minimizing the integral square error (ISE) and is mainly designed for EtherCAT. Its mathematical model assumes that the one-way delay is constant and is measured only once during the network configuration stage. The one-way delay of switched Ethernet is always changing, so optimal PI is unsuitable for switched Ethernet. Later, a Kalman filter (KF) is used in the PI clock servo [28,29,30]. It is a time domain filter with fast response speed and can reduce time offset measurement errors and timestamp quantization errors. Nevertheless, its process and measurement noise covariances, which directly determine the filter performance, are challenging to obtain in practice. Nguyen et al. [33] proposed a fuzzy-PI clock servo that uses fuzzy logic to adjust the system bandwidth online, acquiring faster convergence time and smaller time error. However, it uses the self-tuning method to select fuzzy logic parameters, which is often not optimal. Zhang et al. [34] presented a hybrid control technique based on the improved wolf colony algorithm and cuckoo search algorithm (hybrid IWCA-CS) to optimize fuzzy logic parameters instead of manually adjusting parameters. Their experimental results illustrate that the hybrid IWCA-CS acquires better synchronization performance than the method of Nguyen et al. [33].

As a result, fuzzy-PI is an intelligent control technology recently used in clock servos. It relies on human experience to deal with control problems that are difficult to accurately model. However, it is not explicitly designed for QIDA, and its ability to inhibit QIDA requires further evaluation.

## 3. Background and Problem Statement

### 3.1. Delay Asymmetry Analysis

**Figure 1:**
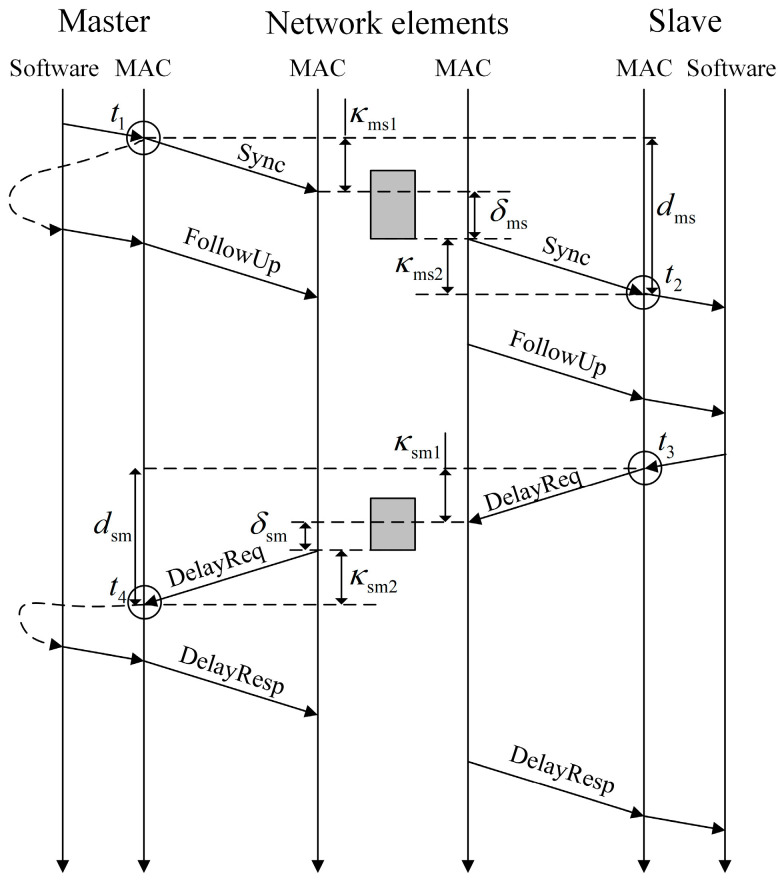
Synchronization process of PTP. Gray squares indicate that BG traffic packets are being transmitted.

The synchronization process of PTP is illustrated in Figure 1. Through four packet exchanges, the slave clock can obtain four timestamps of $t_{1}$, $t_{2}$, $t_{3}$, and $t_{4}$. For details, refer to [9], and the process is repeated every period $T_{\mathrm{sync}}$. For the *n*-th process, assume that x[n] is the time offset (the slave clock time minus the master clock time); $d_{\mathrm{ms}}[n]$ and $d_{\mathrm{sm}}[n]$ represent the master-to-slave (forward) delay and the slave-to-master (backward) delay, respectively. Therefore, the *n*-th time offset can be calculated by

(1) $$\left\{ \begin{array}{l} x[n]=t_{2}[n]-(t_{1}[n]+d_{\mathrm{ms}}[n])\\ x[n]=t_{3}[n]-(t_{4}[n]-d_{\mathrm{sm}}[n]) \end{array}\right..$$

Exchange both sides of Equation (1) and obtain

(2) $$\left\{ \begin{array}{l} t_{21}[n]=t_{2}[n]-t_{1}[n]=x[n]+d_{\mathrm{ms}}[n]\\ t_{43}[n]=t_{4}[n]-t_{3}[n]=-x[n]+d_{\mathrm{sm}}[n] \end{array}\right.$$

where $t_{21}[n]$ and $t_{43}[n]$ are the forward and backward timestamp differences, respectively. Assume that the delay is symmetrical, that is, $d_{\mathrm{ms}}[n]$ and $d_{\mathrm{sm}}[n]$ are equal, and the time offset measurement can be expressed as

(3) $$\tilde{x}[n]=\frac{t_{21}[n]-t_{43}[n]}{2}.$$

Substitute Equation (2) into (3) and obtain

(4) $$\tilde{x}[n]=x[n]+\frac{d_{\mathrm{ms}}[n]-d_{\mathrm{sm}}[n]}{2}=x[n]+w[n]$$

where w[n] is the measurement noise, and it is mainly composed of delay asymmetry, timestamp quantization, and XO frequency drift [44]. As long as the clock frequency is set large enough, the timestamp quantization error is negligible on the μs scale. Moreover, the XO frequency drifts generally slowly and can be considered unchanged in the short term. Therefore, we focus on the effect of delay asymmetry on the measurement. In Figure 1, both $d_{\mathrm{ms}}[n]$ and $d_{\mathrm{sm}}[n]$ can be expressed as the sum of two parts:

(5) $$\left\{ \begin{array}{l} d_{\mathrm{ms}}[n]=\kappa_{\mathrm{ms}1}+\kappa_{\mathrm{ms}2}+\delta_{\mathrm{ms}}[n]=\kappa_{\mathrm{ms}}+\delta_{\mathrm{ms}}[n]\\ d_{\mathrm{sm}}[n]=\kappa_{\mathrm{sm}1}+\kappa_{\mathrm{sm}2}+\delta_{\mathrm{ms}}[n]=\kappa_{\mathrm{sm}}+\delta_{\mathrm{sm}}[n] \end{array}\right..$$

In Equation (5), $\kappa_{\mathrm{ms}}$ and $\kappa_{\mathrm{sm}}$ are static delays, including physical delay and link delay [35]. The sending and receiving delays of the physical layer chip are often different. At the link level, the forward and backward transmission line length and negotiated rate may not be consistent, so the link delay may also be asymmetric. $\delta_{\mathrm{ms}}[n]$ and $\delta_{\mathrm{sm}}[n]$ are the queue delays of the network element, which are dynamic and random [19]. In Figure 1, whenever a Sync packet or a DelayReq packet enters the network element, there may be a BG packet (gray square) being transmitted, especially when the network is congested. Since the lengths of forward and backward BG packets may be different, and the entry moments of the Sync and DelayReq packets are also random, $\delta_{\mathrm{ms}}[n]$ and $\delta_{\mathrm{sm}}[n]$ may be asymmetric. Therefore, the measurement noise w[n] also has static and random components. Substitute Equation (5) into (4) and obtain

(6) $$w[n]=\frac{\kappa_{\mathrm{ms}}-\kappa_{\mathrm{sm}}}{2}+\frac{\delta_{\mathrm{ms}}[n]-\delta_{\mathrm{sm}}[n]}{2}$$

where the left is the static component. Once the network is built, it generally does not change, and asymmetry correction is relatively easy. Moreover, its proportion in w[n] is tiny, almost negligible. The right is the random component, which accounts for a large proportion of w[n]. Consequently, an algorithm must be designed to compensate for QIDA to acquire an accurate time offset estimation.

### 3.2. Frequency Compensation Clock

**Figure 2:**
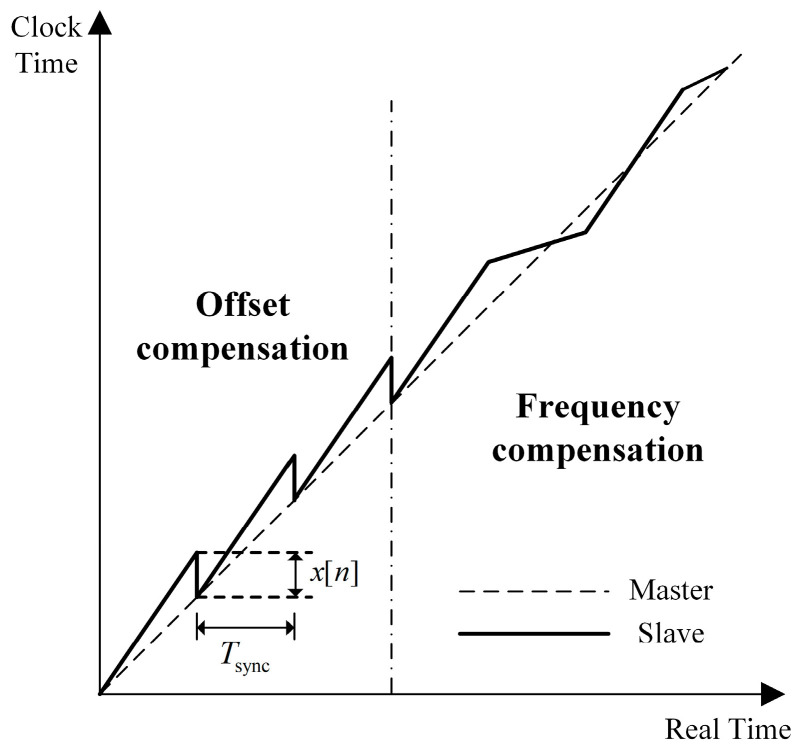
Comparison of two methods for correcting clock time: offset compensation and frequency compensation.

After measuring the time offset, every slave clock corrects the local time and synchronizes with the master clock. There are two main correction methods: offset compensation and frequency compensation [23]. Offset compensation is the slave clock directly adding or subtracting the value based on the local time. In contrast, frequency compensation is the slave clock adjusting the clock rate to eliminate the time offset. The comparison between the two is illustrated in Figure 2. Offset compensation causes the clock time to jump in the opposite direction, while frequency compensation causes the clock time to change continuously and smoothly, so it is adopted by more scholars.

**Figure 3:**
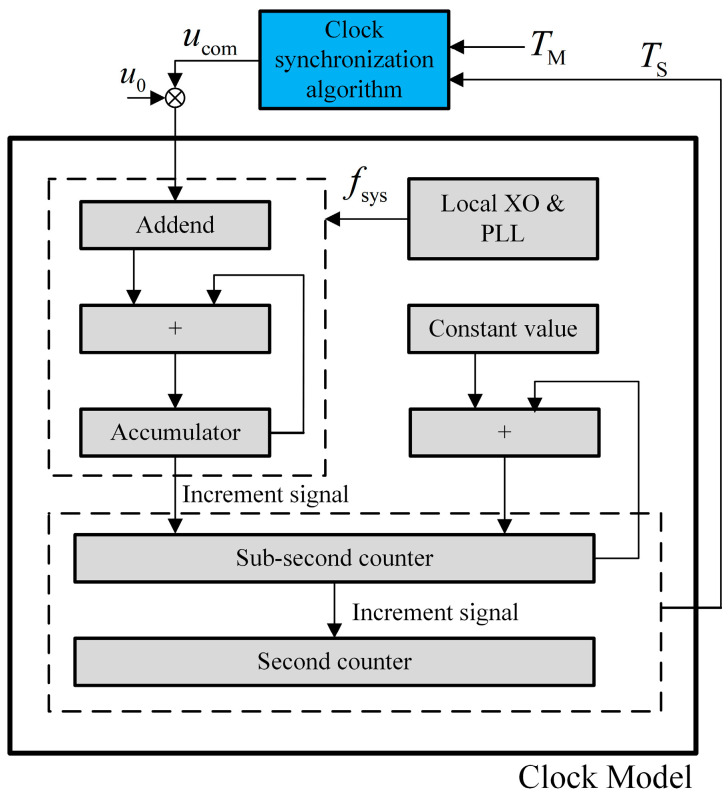
Frequency compensation clock model.

The frequency compensation clock model is demonstrated in Figure 3, which consists of a local XO, an addend, an accumulator, a sub-second counter, and a second counter [45]. The local XO frequency is multiplied by the Phase Locked Loop (PLL) to generate the system clock frequency $f_{\mathrm{sys}}$. The value of the addend is added to the accumulator every system clock cycle. When the accumulator overflows, an increment signal is generated, and the sub-second counter is incremented by a constant value *V*. When the sub-second counter also overflows, the value of the second counter is increased by 1.

The value of the addend is the sum of the initial value $u_{0}$, and the frequency compensation value $u_{\mathrm{com}}$. $u_{\mathrm{com}}$ is updated by the clock synchronization algorithm in each cycle. The calculation method of $u_{0}$ is

(7) $$u_{0}=\frac{2^{32}\cdot f_{0}}{f_{\mathrm{sys}}}$$

where $f_{0}$ represents the slave clock frequency, which is also the overflow frequency of the accumulator. The larger $f_{0}$, the smaller the timing granularity, the smaller the quantization error, and the higher the synchronization accuracy.

The sub-second counter is used to save time of less than 1 s, and the relationship between the increment constant value *V* and the clock frequency $f_{0}$ is

(8) $$f_{0}\cdot V=2^{31}.$$

Eliminate $f_{0}$ according to Equations (7) and (8) and obtain

(9) $$u_{0}=\frac{2^{63}}{f_{\mathrm{sys}}\cdot V}.$$

To sum up, the addend directly affects the overflow frequency of the accumulator, which essentially determines the slave clock frequency. Selecting an appropriate algorithm to calculate the frequency compensation value can control the slave clock frequency and ensure accurate synchronization of the master and slave clocks.

## 4. Design of the Clock Servo

**Figure 4:**
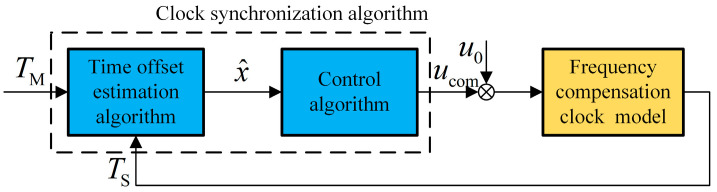
Composition of the clock servo system.

As established in Section 3, the synchronization algorithm has two goals: one is to obtain an accurate time offset estimation under QIDA, and the other is to provide an appropriate frequency compensation value. The first goal requires designing a time offset estimation algorithm. This paper uses a packet selection algorithm based on oversampling, and the selection strategy uses the minimum. The second goal requires a control algorithm. If the estimation result of the packet selection algorithm is directly used for compensation, the time error is prone to large fluctuations, and the accuracy is not high. The control algorithm adopts the PI controller, and we add fuzzy control to adaptively adjust PI coefficients so that the system can converge quickly and have some anti-noise performance after stabilization. The time offset estimation algorithm and the control algorithm, combined with the frequency compensation clock model, can constitute a complete clock servo system, as shown in Figure 4.

### 4.1. Minimum Window Filter

The sampling period of the clock servo is also the synchronization period, and the default value is 1 s. Since the estimation algorithm adopts oversampling, the sampling speed needs to be increased by about ten times to ensure sufficient samples. Although oversampling will increase the number of PTP packets in the network and the processing burden of the master and slave clocks, a balance point can be found as long as the sampling period is adequately designed. The packet selection algorithm is essentially a window filter. When the number of PTP packets reaches the window length, the minimum delay packets are searched in the master-to-slave and slave-to-master directions. The queue delays of these two packets are negligible ($\delta_{\mathrm{ms}}\approx \delta_{\mathrm{sm}}\approx 0$), and we substitute them into Equation (6), thus having the slightest measurement noise. The data exchanged between nodes in a DMCS are mostly measurement and control information. The information density is not high, and the actual BG traffic is generally less than 50% [19]. The smaller the BG traffic, the higher the probability of finding the minimum delay packet. Therefore, it is more appropriate to use the minimum strategy in this scenario.

Assume that the length of the observation window is *N*, and each window does not overlap. In the *k*-th window, the timestamp difference vectors can be expressed as

(10) $$\left\{ \begin{array}{l} t_{21}[k]=[t_{21}{\left[k,0],t_{21}[k,1],\cdot \cdot \cdot ,t_{21}[k,N-1]\right]}^{T}\\ t_{43}[k]=[t_{43}{\left[k,0],t_{43}[k,1],\cdot \cdot \cdot ,t_{43}[k,N-1]\right]}^{T} \end{array}\right.$$

where $t_{21}[k,m](m\in [0,N-1])$ represents the *m*-th timestamp difference in the *k*-th window. Because this paper uses frequency compensation to correct the time offset, there must be a frequency offset between the master and slave clocks, which will cause $t_{21}[k]$ and $t_{43}[k]$ to drift. Therefore, drift compensation must be implemented before packet selection.

**Figure 5:**
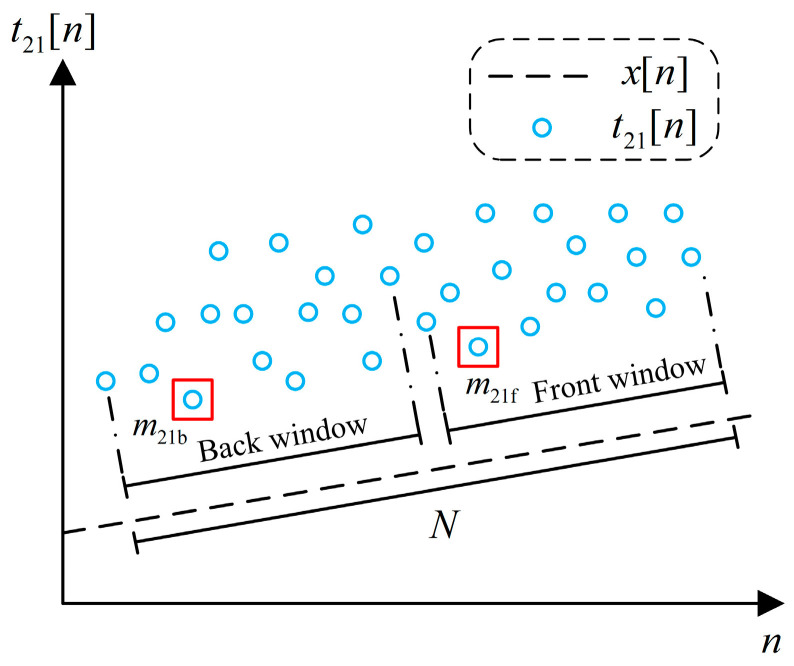
One-way estimation method of the frequency offset using $t_{21}[k]$. Circles in two red boxes indicate the minimum timestamp differences inside the front and back windows.

The first step of drift compensation is to estimate the frequency offset in the window, and the one-way estimation method using $t_{21}[k]$ is presented in Figure 5. The dotted line in the figure indicates the true time offset, and the circle indicates the timestamp difference. The vertical distance between the two represents the delay, according to Equation (2). Divide the window into the front and back parts, find the minimum timestamp differences, respectively, and record their indexes $m_{21f}$ and $m_{21b}$. The one-way frequency offset estimation can be described as

(11) $${\hat{y}}_{21}[k]=\frac{\underset{0\leq j<N/2}{\psi }\{t_{21}[k,N/2+j]\}-\underset{0\leq j<N/2}{\psi }\{t_{21}[k,j]\}}{m_{21f}+N/2-m_{21b}}$$

where ψ{} represents an operator, which can be the minimum, maximum, median, etc. This paper uses the minimum. Similarly, the frequency offset estimation in another direction using $t_{43}[k]$ can be denoted as ${\hat{y}}_{43}[k]$. If the minimum delay packets can be found in both the front and the back windows, ${\hat{y}}_{21}[k]$ and ${\hat{y}}_{43}[k]$ will have the same absolute value and opposite signs in theory. On the contrary, if the packets cannot be found, ${\hat{y}}_{21}[k]$ or ${\hat{y}}_{43}[k]$ may have significant variation. To reduce the estimation error, the final frequency offset estimation takes the smaller absolute value of the two, and the sign is consistent with ${\hat{y}}_{21}[k]$, which can be expressed as

(12) $$\hat{y}[k]=\{\begin{array}{cc} {\hat{y}}_{21}[k] & \left|{\hat{y}}_{21}[k]\right|\leq \left|{\hat{y}}_{43}[k]\right|\\ -{\hat{y}}_{43}[k] & \left|{\hat{y}}_{21}[k]\right|>\left|{\hat{y}}_{43}[k]\right| \end{array}.$$

Compared with ref. [35], we simultaneously use samples from two directions to estimate the frequency offset, effectively reducing estimation error. Moreover, the estimation process does not need to construct a second window. Since the synchronization period $T_{\mathrm{sync}}$ is constant in this paper, there is no need to substitute $T_{\mathrm{sync}}$ when calculating ${\hat{y}}_{21}[k]$ or ${\hat{y}}_{43}[k]$. So, the unit of $\hat{y}[k]$ is $s/T_{\mathrm{sync}}$, which is easy for subsequent calculation. We use $\hat{y}[k]$ to perform drift compensation and correct vectors $t_{21}[k]$ and $t_{43}[k]$:

(13) $$\left\{ \begin{array}{l} t_{21}^{\prime }[k,m]=t_{21}[k,m]-\hat{y}[k]\cdot (m+1)\\ t_{43}^{\prime }[k,m]=t_{43}[k,m]+\hat{y}[k]\cdot (m+1) \end{array}\right..$$

The final time offset estimation can be obtained by

(14) $$\hat{x}[k]=\frac{\psi \{t_{21}^{\prime }[k]\}-\psi \{t_{43}^{\prime }[k]\}}{2}+\hat{y}[k]\cdot N.$$

The premise of using the minimum window filter with drift compensation is that the frequency offset remains constant within the window, which is a loose constraint and allows a longer window. Algorithm 1 details the above time offset estimation process.

**Algorithm 1:** Time offset estimation using the minimum window filter

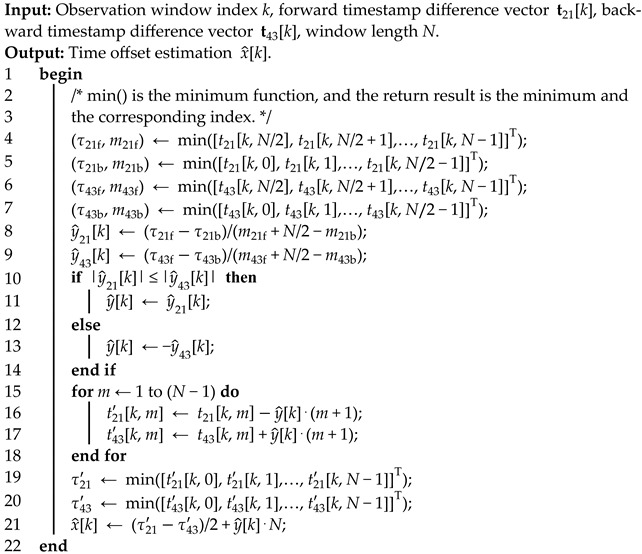



### 4.2. Fuzzy-PI Controller

The minimum window filter is an oversampling nonlinear filter. Since the observation window is non-overlapping, the frequency correction period $T_{c}$ of the clock servo is magnified by *N* times compared to the synchronization period $T_{\mathrm{sync}}$:

(15) $$T_{c}=NT_{\mathrm{sync}}.$$

**Figure 6:**
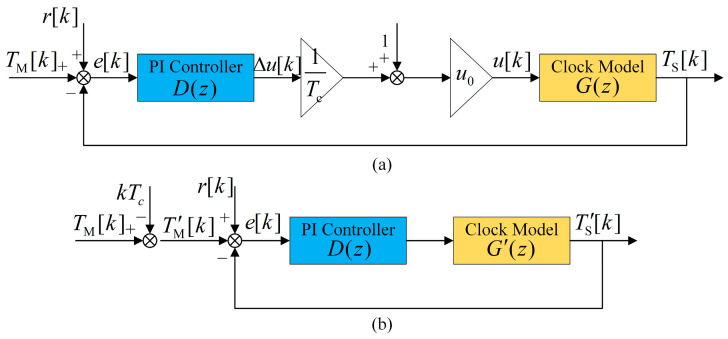
Control system model of the clock servo (ignore the fuzzy logic). (**a**) Original diagram. (**b**) Simplified diagram after constructing a new input and output.

We temporarily ignore the fuzzy logic and establish the control system model of the clock servo after window filtering, as observed in Figure 6a. $T_{M}[k]$ and $T_{S}[k]$ are the master and slave clock times. e[k] is the time offset estimation $\hat{x}[k]$, and r[k] is the remaining estimation error after window filtering. D(z) is the transfer function (TF) of the PI controller, which can be described as

(16) $$D(z)=k_{p}+k_{i}\frac{z}{z-1}$$

where $k_{p}$ and $k_{i}$ are PI coefficients. ∆u[k] is the adjustment value output by the PI controller, and u[k] is the value of the addend in Figure 3. G(z) is the clock TF, and the expression is

(17) $$G(z)=\frac{k_{c}T_{c}}{z-1}$$

where $k_{c}$ is a constant coefficient, which can be expressed as

(18) $$k_{c}=\frac{f_{\mathrm{sys}}V}{2^{63}}.$$

The clock model’s input u[*k*] consists of two parts, which are summed through the comparison point. We move the comparison point backward and construct a new input $T_{M}^{\prime }[k]$ and output $T_{S}^{\prime }[k]$:

(19) $$\left\{ \begin{array}{l} T_{S}^{\prime }[k]=T_{S}[k]-kk_{c}u_{0}T_{c}=T_{S}[k]-kT_{c}\\ T_{M}^{\prime }[k]=T_{M}[k]-kk_{c}u_{0}T_{c}=T_{M}[0]+kT_{c}-kT_{c}=T_{M}[0] \end{array}\right..$$

The calculation process can refer to Equations (9) and (18), and $T_{M}\left[0\right]$ is the initial value of the master clock time. Combining the clock model with the two coefficients in front results in the simplified diagram shown in Figure 6b. The new clock TF is

(20) $$G^{\prime }(z)=\frac{1}{z-1}.$$

The time offset e[k] values before and after simplification are entirely equivalent. The original input $T_{M}[k]$ is a ramp input, and the new input $T_{M}^{\prime }[k]$ is a step input from Equation (19). The closed-loop TF of the system can be expressed as

(21) $$\Phi (z)=\frac{T_{s}^{\prime }(z)}{T_{M}^{\prime }(z)}=\frac{G^{\prime }(z)D(z)}{1+G^{\prime }(z)D(z)}=\frac{(k_{p}+k_{i})z-k_{p}}{z^{2}+(k_{p}+k_{i}-2)z+(1-k_{p})}.$$

The system is a second-order discrete system. We need to find a method to determine PI coefficients. The general closed-loop TF of a second-order continuous system can be expressed as

(22) $$\Phi_{s}(s)=\frac{2\xi \omega_{n}s+\omega_{n}^{2}}{s^{2}+2\xi \omega_{n}s+\omega_{n}^{2}}$$

The system poles are

(23) $$s_{1,2}=-\xi \omega_{n}\pm j\omega_{n}\sqrt{1-\xi^{2}}=-\xi \omega_{n}\pm j\omega_{d}$$

where ξ is the damping ratio, $\omega_{n}$ is the natural frequency, and $\omega_{d}$ is the damped frequency. According to the z-transform, the poles of the corresponding discrete system can be obtained as follows:

(24) $$z_{1,2}=e^{s_{1,2}T_{c}}=e^{-\xi \omega_{n}T_{c}}e^{\pm j\omega_{d}T_{c}}=e^{-\xi \omega_{n}T_{c}}∠\pm \omega_{d}T_{c}.$$

According to Equation (24), the general characteristic polynomial of the second-order discrete system can be expressed as

(25) $$\begin{array}{l} P(z)=(z-z_{1})(z-z_{2})=(z-e^{-\xi \omega_{n}T_{c}}e^{j\omega_{d}T_{c}})(z-e^{-\xi \omega_{n}T_{c}}e^{-j\omega_{d}T_{c}})\\ =z^{2}-(2\cos(\omega_{d}T_{c}))e^{-\xi \omega_{n}T_{c}}z+e^{-2\xi \omega_{n}T_{c}}. \end{array}$$

Equation (25) should be equal to the corresponding coefficients of the denominator of (21), and the expressions of $k_{p}$ and $k_{i}$ can be acquired as follows:

(26) $$\left\{ \begin{array}{l} k_{p}=1-e^{-2\xi \omega_{n}T_{c}}\\ k_{i}=1-2(\cos(\omega_{d}T_{c}))e^{-\xi \omega_{n}T_{c}}+e^{-2\xi \omega_{n}T_{c}} \end{array}\right..$$

PI coefficients are related to three parameters: the frequency correction period $T_{c}$, damping ratio ξ, and natural frequency $\omega_{n}$. The equivalent noise bandwidth of a second-order system can be expressed as [46]

(27) $$B_{L}=\frac{1}{2\pi }\int_{0}^{\infty }{\left|\Phi_{s}(j\omega )\right|}^{2}d\omega =\frac{\omega_{n}}{2}(\xi +\frac{1}{4\xi }).$$

When the damping ratio ξ is fixed, the system bandwidth $B_{L}$ is proportional to the natural frequency $\omega_{n}$. The larger $\omega_{n}$ and the wider $B_{L}$, the stronger the system’s dynamic performance, which is suitable for the initial unstable stage to ensure rapid convergence. On the contrary, the smaller $\omega_{n}$ and the narrower $B_{L}$, the weaker the dynamic performance, but the loop exhibits low-pass characteristics and has a strong ability to suppress input noise, which is suitable for the synchronization stabilization stage. The relationship between the time offset and the bandwidth is challenging to model, but it can be described with a series of language rules, so we use fuzzy control.

**Figure 7:**
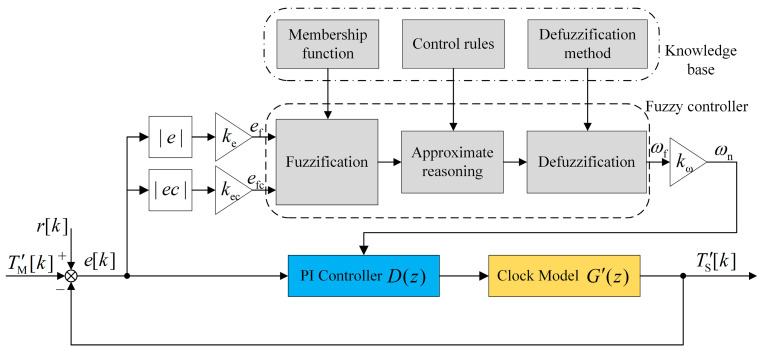
Schematic diagram of the fuzzy-PI clock servo.

The schematic diagram of the clock servo after adding fuzzy control is demonstrated in Figure 7. The absolute time offset |e| and absolute offset derivative |ec| are the two inputs of the fuzzy controller, and the natural frequency $\omega_{n}$ is its output. $k_{e}$ and $k_{\mathrm{ec}}$ are input scaling factors, and $k_{\omega }$ is the output scaling factor. They are used to separate physical and fuzzy domains and improve the design flexibility. The fuzzy controller is composed of three parts: fuzzification, approximate reasoning, and defuzzification, as shown in the dotted line box in Figure 7. Moreover, the fuzzy process requires using several modules in the knowledge base: membership function (MF), control rules, and defuzzification method. The fuzzification module converts the input from numeric values into fuzzy sets, and then the approximate reasoning module performs logical operations based on control rules to acquire fuzzy values. Finally, the defuzzification module converts the fuzzy values into accurate numeric values.

Assume that the physical domain of |e| is [0,E] and the fuzzy domain is $\left[-E_{f},E_{f}\right]$. The physical domain of |ec| is $[0,E_{c}]$ and the fuzzy domain is $\left[-E_{\mathrm{fc}},\;E_{\mathrm{fc}}\right]$. The physical domain of $\omega_{n}$ is $\left[\Omega_{d},\Omega_{u}\right]$ and the fuzzy domain is $\left[{-\Omega }_{f},\Omega_{f}\right]$. The input scaling factor $k_{e}$ of |e| can be expressed as

(28) $$k_{e}=\frac{2E_{f}}{E}.$$

**Figure 8:**
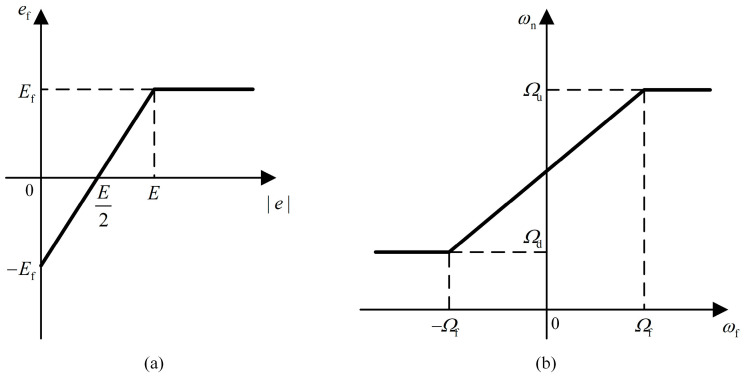
Mapping function between the fuzzy and physical domains of the fuzzy controller. (**a**) Absolute time offset |e|. (**b**) Natural frequency $\omega_{n}$.

The mapping function between its fuzzy and physical domains is as follows, and the function plot is illustrated in Figure 8a.

(29) $$e_{f}=\{\begin{array}{cc} k_{e}(|e|-\frac{E}{2}) & 0\leq |e|<E\\ E_{f} & |e|\geq E \end{array}.$$

The scaling factor and mapping function of |ec| are the same as above. The output scaling factor $k_{\omega }$ of $\omega_{n}$ can be expressed as

(30) $$k_{\omega }=\frac{\Omega_{u}-\Omega_{d}}{2\Omega_{f}}.$$

The corresponding mapping function of $\omega_{n}$ is as follows, and the function plot is also shown in Figure 8b.

(31) $$\omega_{n}=\{\begin{array}{cc} \Omega_{d} & \omega_{f}\leq -\Omega_{f}\\ k_{\omega }(\omega_{f}+\frac{\Omega_{f}(\Omega_{u}\;+\;\Omega_{d})}{\Omega_{u}\;-\;\Omega_{d}}) & -\Omega_{f}<\omega_{f}<\Omega_{f}\\ \Omega_{u} & \omega_{f}\geq \Omega_{f} \end{array}.$$

**Figure 9:**
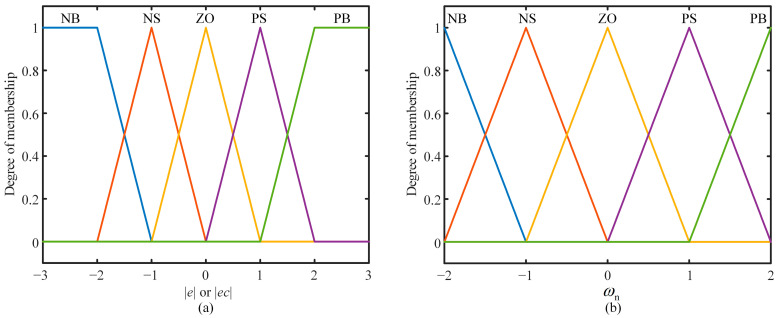
Membership function. (**a**) Absolute time offset |e| and absolute offset derivative |ec|. (**b**) Natural frequency $\omega_{n}$.

In order to facilitate calculation and ensure control accuracy, the input and output fuzzy sets are all set to five: NB, NS, ZO, PS, and PB. The MF uses a triangle to ensure a smaller computational burden and higher resolution. The absolute time offset |e| and absolute offset derivative |ec| have the same MF, as shown in Figure 9a, and the boundary of the fuzzy domain $E_{f}=E_{\mathrm{fc}}=3$. The MF of the natural frequency $\omega_{n}$ is shown in Figure 9b, and the boundary $\Omega_{f}=2$. The approximate reasoning method uses the Mamdani algorithm. For different inputs, the output should meet the following human experience:

- If |e| and |ec| are large, a large $\omega_{n}$ should be selected to speed up the system response and ensure rapid convergence.
- If |e| and |ec| are moderate, a moderate $\omega_{n}$ should be chosen so that the system has smaller overshoot.
- If |e| and |ec| are small, a small $\omega_{n}$ should be taken so that the system has good steady-state performance and anti-noise ability.

According to the above experience, fuzzy control rules are generated, as illustrated in Table 3, with a total of twenty-five rules. In addition, the defuzzification method adopts the center of gravity method. Using the output of the fuzzy controller, the corresponding PI coefficients can be calculated according to Equation (26).

The largest difference between our clock servo and the fuzzy-PI servo [33,34] is the introduction of a window filter for compensating QIDA, meaning that our servo performs better in asymmetric networks (please refer to Section 6.2). Moreover, the clock servo is essentially a discrete system, and we build a more accurate discrete model to adjust PI coefficients compared to refs. [33,34].

## 5. Experimental Platform

### 5.1. System Introduction

**Figure 10:**
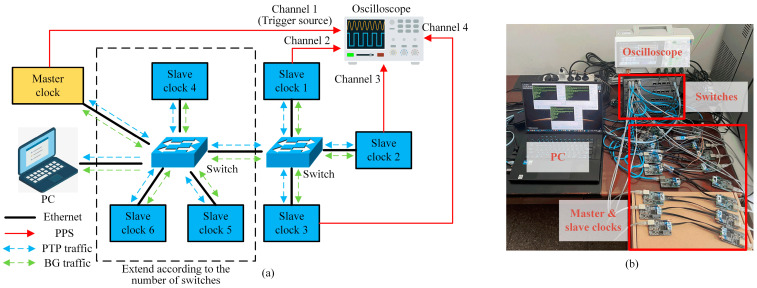
Experimental platform. (**a**) Schematic diagram. (**b**) Physical diagram.

To test the performance of the clock servo proposed in Section 4, we construct the experimental platform illustrated in Figure 10a. The simplest system is a single-hop system, where one master clock, three slave clocks, and one personal computer (PC) are directly connected to one switch through network cables. The switch uses S5735S-L8T4S-QA2 (nearly USD 100 per switch) (Huawei, Shenzhen, China), and the PC is used to monitor traffic changes in the network. The oscilloscope uses ZLG ZDS1104 (Zhiyuan Electronics, Guangzhou, China), which is used to observe the time error fluctuation. Connect the Pulse Per Second (PPS) signals of the master clock and slave clocks 1, 2, and 3 to the four channels of the oscilloscope, and set the PPS signal of the master clock (channel 1) as the trigger source. The system scale can be expanded based on the single-hop system. Every time one switch is added, three slave clocks are directly connected to this switch, and the master clock and PC are also transferred to this switch. The oscilloscope still monitors the master clock and slave clocks 1, 2, and 3. The expansion method is observed in the dotted line box in Figure 10a, and Figure 10b is a physical diagram according to Figure 10a.

The master and slave clocks use the same nodes, and the hardware cost of each node is within USD 10. The node microcontroller unit (MCU) chooses STM32F407VGT6 (STMicroelectronics, Geneva, Switzerland), and the system frequency is set to 168 MHz. Its integrated Ethernet controller can obtain hardware timestamps at the MAC layer. The physical layer chip uses LAN8720A (Microchip, Chandler, AZ, USA). The XO uses KDS DSX321G (Daishinku, Kakogawa, Japan), the nominal frequency is 8 MHz, the frequency tolerance is ±20 ppm (25 °C), and the frequency stability is ±50 ppm (−40 to +105 °C). The node software uses the free real-time operating system (FreeRTOS) and is further developed based on the open-source PTP Daemon project [47].

### 5.2. BG Traffic Model

Most scholars used two traffic models when studying QIDACM: cross-traffic and in-line traffic [38]. Unlike the above two models, we use a broadcast traffic model, which is more suitable for a DMCS. The master and slave clocks can generate PTP traffic and BG traffic simultaneously, and the transmission paths of the two are the same, as observed regarding the blue and green dotted lines in Figure 10a. This model is particularly suitable for one-to-many communication. Each node can decide whether to receive the BG traffic packet based on the internal filter, which is convenient for system scalability and redundancy design. This traffic model is widely used in EtherNet/IP for industrial automation. The BG traffic packet is transmitted periodically using the UDP multicast protocol. Unless otherwise stated, the BG traffic in this article refers to the total traffic. The BG traffic generated by each clock is equally distributed according to the number of clocks. For example, assume that the total BG traffic is 60 Mbps and the single-hop system has four clocks, so each clock needs to generate 15 Mbps BG traffic. In addition, we do not use Virtual Local Area Network (VLAN) to set priorities for PTP packets and BG traffic packets to improve the applicability of our clock servo.

## 6. Results and Discussion

### 6.1. Effect of Different Parameters on Synchronization Performance

This subsection studies the effect of different parameters on synchronization performance, which mainly includes algorithm parameters and external parameters, as presented in Table 4. The algorithm parameters comprise slave clock period $T_{0}$, synchronization period $T_{\mathrm{sync}}$, observation window length *N*, and PI coefficients $k_{p}$ and $k_{i}$. The slave period $T_{0}$ is the reciprocal of the slave clock frequency $f_{0}$. The smaller the value of $T_{0}$, the larger the value of $f_{0}$ and the smaller the quantization error. $T_{0}$ is set to 7 ns, and then, according to Equations (8) and (9), the constant value *V* and the initial value $u_{0}$ of the addend are 15 and 0xDA2835AC. IEEE 1588 standard stipulates that the minimum synchronization period is 7.8125 ms [9]. The smaller the value of $T_{\mathrm{sync}}$, the better the synchronization performance, but the greater the pressure on the clocks to process PTP packets. Therefore, the compromise is to set $T_{\mathrm{sync}}$ to 125 ms. The window length *N* is set to 32, ensuring enough samples for estimation. It is a power of two and convenient for shift calculation. *N* should not be too large because, the larger *N*, the longer the frequency correction period $T_{c}$, and the easier it is for time error to accumulate. PI coefficients $k_{p}$ and $k_{i}$ are calculated by the fuzzy controller in each correction period. From Section 4.2, the physical domain range of the fuzzy controller has not been determined, which will be studied later.

The external parameters comprise BG traffic $W_{\mathrm{bg}}$, BG traffic packet length $L_{\mathrm{bg}}$, switch hop count $N_{\mathrm{hop}}$, and ambient temperature $T_{\mathrm{temp}}$. Since the node hardware only supports 100 Mbps Ethernet, the BG traffic $W_{\mathrm{bg}}$ ranges from 0 to 100 Mbps. The BG traffic packet length $L_{\mathrm{bg}}$ has three values: 512, 1024, and 1518 Bytes (1518 is the maximum frame length of Ethernet). The larger the value of $L_{\mathrm{bg}}$, the longer the queue delay for PTP packets to collide with BG traffic packets. The switch hop count $N_{\mathrm{hop}}$ supports from 1 to 5, which is already the size of medium networks. All the experiments are carried out at room temperature for convenience.

#### 6.1.1. PI Coefficients

**Figure 11:**
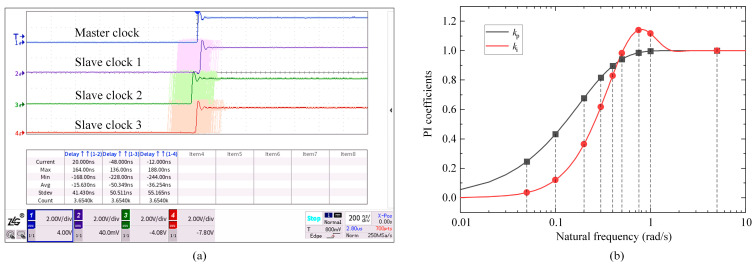
(**a**) Time error fluctuation recorded by the oscilloscope for one hour (damping ratio is 0.707, and natural frequency is 0.2 rad/s). Oscilloscope parameter settings: horizontal time scale 0.2 μs/div, vertical voltage scale 2 V/div, rising edge trigger, trigger level 0.8 V, and afterglow mode enabled. (**b**) PI coefficients for varying natural frequency (damping ratio is 0.707, and frequency correction period is 4 s). Intersections of dashed lines and solid lines indicate PI coefficients used for the experiments.

First, we study the effect of PI coefficients $k_{p}$ and $k_{i}$ on synchronization performance. The experimental parameters are set as follows. The BG traffic $W_{\mathrm{bg}}$ is 50 Mbps, the BG traffic packet length $L_{\mathrm{bg}}$ is 1518 Bytes, and the switch hop count $N_{\mathrm{hop}}$ is one. Fuzzy control is temporarily ignored, and $k_{p}$ and $k_{i}$ are artificially provided during initialization and remain unchanged during the experiment. Set the damping ratio ξ to 0.707 (the best value in engineering), the natural frequency $\omega_{n}$ to 0.2 rad/s, and the corresponding $k_{p}$ and $k_{i}$ are 0.677 and 0.364, respectively, for the experiments. First, record the convergence time of the synchronization algorithm. The initial time offset is set to 1 ms, and the convergence condition is that the absolute time error (|TE|) is less than 1 μs. Then, enable the afterglow mode of the oscilloscope to record the time error fluctuation for one hour, and the result is shown in Figure 11a. The mean time error values between slave clocks 1, 2, and 3 and the master clock are −0.016 μs, −0.050 μs, and −0.036 μs. The standard deviations (STDs) are 0.041 μs, 0.051 μs, and 0.055 μs. The max |TE| values are 0.168 μs, 0.228 μs, and 0.244 μs.

**Figure 12:**
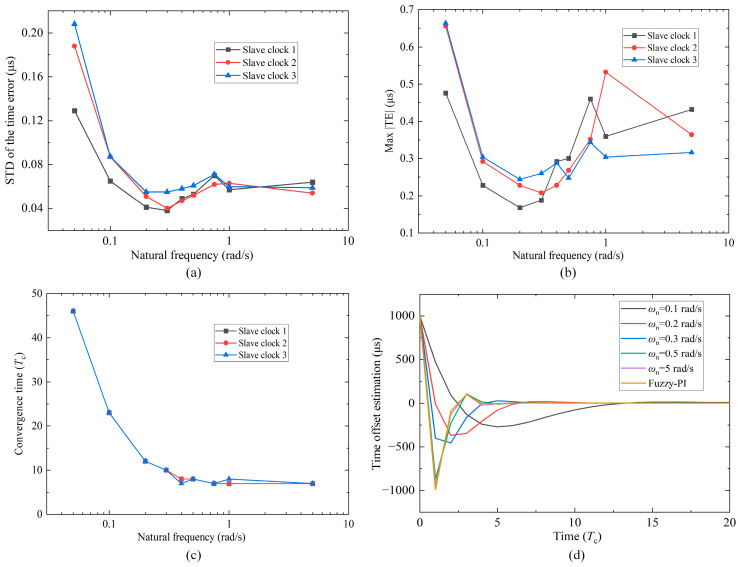
Results of the PI coefficients experiment. (**a**) STD of the time error for varying natural frequency. (**b**) Max |TE| for varying natural frequency. (**c**) Convergence time for varying natural frequency. (**d**) Convergence process of the time offset (slave clock 2; initial offset 1 ms; convergence condition |TE| ≤ 1 μs).

In order to study the performance under different natural frequencies, $\omega_{n}$ is selected as 0.05, 0.1, 0.3, 0.4, 0.5, 0.75, 1, and 5 rad/s, and the relationship between the selected values of $\omega_{n}$ and the PI coefficients is shown in Figure 11b. Multiple experiments are carried out, and the results are presented in Table 5. Variations in the STD of the time error and the max |TE| with natural frequency are demonstrated in Figure 12a,b. If $\omega_{n}$ is too small ($\omega_{n}$ is less than 0.1 rad/s), although the loop’s ability to suppress input noise becomes stronger, the dynamic performance becomes weaker, and the jitter of the XO frequency will cause large time error fluctuation. Both the STD and the max |TE| increase significantly. On the contrary, if $\omega_{n}$ is too large ($\omega_{n}$ is greater than 0.5 rad/s), the loop’s ability to suppress input noise becomes weaker, and the max |TE| increases significantly. Therefore, $\omega_{n}$ has an optimal intermediate value of 0.2 or 0.3 rad/s. When $\omega_{n}$ is 0.3 rad/s, the STD values of the time error of slave clocks 1, 2, and 3 are 0.038 μs, 0.040 μs, and 0.055 μs, achieving the global minimum. The max |TE| values are 0.188 μs, 0.208 μs, and 0.260 μs. The reason why the STD of slave 3 is obviously larger than that of slaves 1 and 2 may be that the stability of its XO frequency at room temperature is poor.

Table 5 also provides the algorithm convergence time. The variation in the convergence time with $\omega_{n}$ is shown in Figure 12c. Figure 12d displays the convergence process of the time offset of slave clock 2. The larger the value of $\omega_{n}$, the faster the convergence speed, but overshoot will increase. When $\omega_{n}$ is 5 rad/s, both $k_{p}$ and $k_{i}$ reach the stable value of 1.000. Substitute them into Equation (21), and the two poles of the system are located at the origin, so the dynamic performance is the best, and the convergence time only takes seven correction periods.

Based on the above results, the physical domain range of the fuzzy controller can be determined. Since the synchronization accuracy target in this paper is 1 μs, the upper bound E of the absolute time offset |e| is also set to 1 μs. Since the absolute offset derivative |ec| values when $\omega_{n}$ is 0.2 or 0.3 rad/s are both within 0.06 μs/s, the upper bound $E_{c}$ of |ec| is also set to 0.06 μs/s. Moreover, the upper bound $\Omega_{u}$ and lower bound $\Omega_{d}$ of the natural frequency $\omega_{n}$ are 0.6 rad/s (better dynamic performance) and 0.2 rad/s (optimal intermediate value). The results after adding fuzzy control are presented in the last row of Table 5. The STD values of the time error of slave clocks 1, 2, and 3 are 0.039 μs, 0.046μs, and 0.057μs, and the max |TE| values are 0.168 μs, 0.248 μs, and 0.196 μs. These results are relatively close to those when $\omega_{n}$ is 0.2 or 0.3 rad/s. The convergence time is seven to eight correction periods, about half a minute, almost reaching the fastest speed. As a result, fuzzy-PI can ensure fast convergence and obtain good synchronization performance simultaneously. All the subsequent experiments use the above fuzzy-PI controller.

#### 6.1.2. BG Traffic

**Figure 13:**
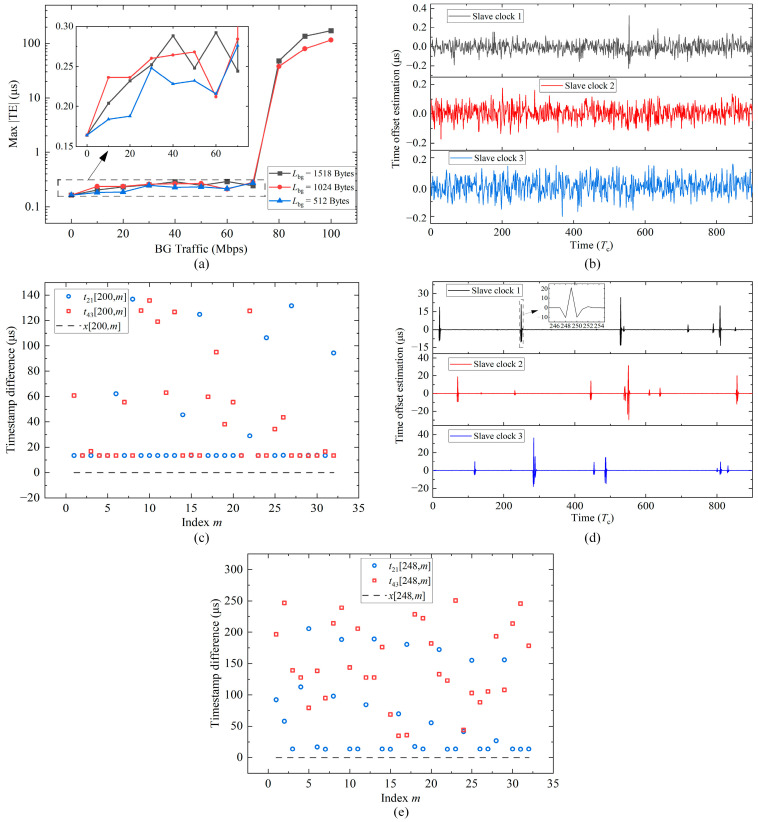
Results of the BG traffic experiments. (**a**) Max |TE| for varying BG traffic. (**b**) Time offset estimation recorded for one hour when BG traffic is 40 Mbps and length is 1518 Bytes. (**c**) Forward and backward timestamp differences from the 200-th window of slave clock 1 in (**b**). (**d**) Time offset estimation when BG traffic is 80 Mbps and length is 1518 Bytes. (**e**) Forward and backward timestamp differences from the 248-th window of slave clock 1 in (**d**).

**Figure 14:**
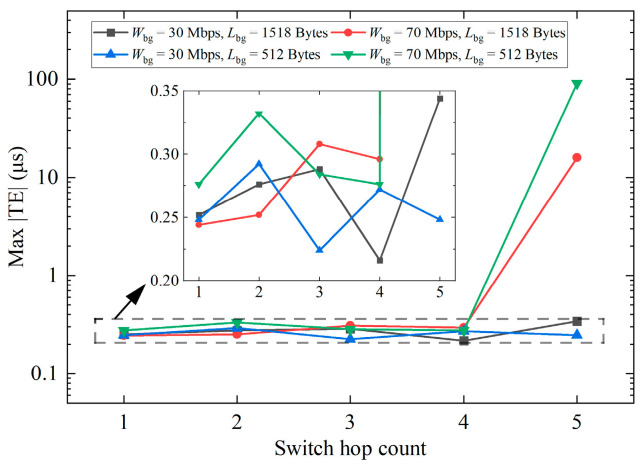
Max |TE| for varying switch hop count.

Subsequently, BG traffic experiments are conducted, and the switch hop count remains one. The BG traffic $W_{\mathrm{bg}}$ ranges from 0 to 100 Mbps. The results are illustrated in Figure 13a. The vertical axis means the maximum among the max |TE| of slave clocks 1, 2, and 3. The vertical axes in Figure 14 and Figure 16 have the same meaning. When there is no BG traffic, the max |TE| is 0.164 μs. When BG traffic is added and controlled within 70 Mbps, the max |TE| shows an increasing trend, but the change range is not large and remains within 0.3 μs. However, the greater the value of $W_{\mathrm{bg}}$, the greater the probability that PTP packets will be affected by queue delay. Nevertheless, as long as the minimum delay packets can be found within the window, the time offset estimation using Algorithm 1 can be close to the true value, and the synchronization accuracy can be guaranteed. Figure 13b shows the time offset estimation when $W_{\mathrm{bg}}$ is 40 Mbps and the length $L_{\mathrm{bg}}$ is 1518 Bytes, and the estimation value is basically within 0.3 μs. Figure 13c shows the forward and backward timestamp differences from the 200-th window of slave clock 1 in Figure 13b. The dotted line indicates the true time offset, which is close to zero. The vertical distance between the circles or boxes and the dotted line represents the delay. Most PTP packets are not affected by queue delays, and the forward and backward static delays $\kappa_{\mathrm{ms}}$ and $\kappa_{\mathrm{sm}}$ are about 13.4 μs. Therefore, the algorithm estimation accuracy is excellent, and the result is 0.051 μs. When $L_{\mathrm{bg}}$ is 512 Bytes, the maximum of $W_{\mathrm{bg}}$ is only 70 Mbps. Because of the smaller $L_{\mathrm{bg}}$, in order to achieve the same BG traffic, the number of packets will be greater, and the software will have more overhead in packing and unpacking packets. When $W_{\mathrm{bg}}$ is 70 Mbps, the MCU utilization is measured to exceed 90%. If $W_{\mathrm{bg}}$ continues to increase, the MCU will not have enough time to process PTP packets.

When $W_{\mathrm{bg}}$ is increased to 80 Mbps and $L_{\mathrm{bg}}$ is 1024 or 1518 Bytes, the max |TE| values are 47.8 μs and 37.8 μs, respectively. Figure 13d includes the time offset estimation when $W_{\mathrm{bg}}$ is 80 Mbps and $L_{\mathrm{bg}}$ is 1518 Bytes. The offset has multiple jumps, meaning that the packet selection algorithm begins to fail. Figure 13e shows the forward and backward timestamp differences from the 248-th window of slave clock 1 in Figure 13d. Compared to Figure 13c, its PTP packets affected by queue delays increase significantly, and all the red boxes are affected, so the estimation deviates from the true value and is −10.484 μs. This value is obviously a gross estimation point, and secondary filtering can be used to eliminate it later. Thanks to the excellent dynamic performance of fuzzy-PI, the time offset can return to normal with five to six correction periods. When $W_{\mathrm{bg}}$ increases to 90 and 100 Mbps, the synchronization performance continues to deteriorate, and the accuracy can only reach a hundred microseconds.

To sum up, our clock servo can guarantee 1 μs synchronization accuracy within 70 Mbps BG traffic, which meets the needs of most scenarios.

#### 6.1.3. Switch Hop Count

Finally, the effect of switch hop count $N_{\mathrm{hop}}$ on synchronization performance is studied. The system expansion method is described in Section 5.1. As $N_{\mathrm{hop}}$ increases, the number of clocks increases, and the output traffic on the switch ports directly connected to each clock also increases. At the same time, the communication paths between the master clock and slave clocks 1, 2, and 3 become longer, and the uncertainty of PTP packets colliding with BG packets will also increase. The experimental result is illustrated in Figure 14. The BG traffic $W_{\mathrm{bg}}$ is set to 70 or 30 Mbps, and the length $L_{\mathrm{bg}}$ is set to 1518 or 512 Bytes. When $N_{\mathrm{hop}}$ is within four, the variation in $N_{\mathrm{hop}}$ will not affect synchronization performance, and the max |TE| fluctuates within 0.35 μs. When $N_{\mathrm{hop}}$ is increased to five and $W_{\mathrm{bg}}$ is 70 Mbps, we observed that the packet selection algorithm began to fail occasionally, similar to Figure 13d, and the max |TE| reaches tens of microseconds. When $W_{\mathrm{bg}}$ is 30 Mbps, the max |TE| can be guaranteed to less than 0.35 μs.

Ref. [19] also contains the experimental results regarding switch hop count. Using TC switches, the max |TE| of single-hop, two-hop, and three-hop can all be kept around 0.03 μs, and the accuracy is very high. If ordinary switches are used, the performance will obviously deteriorate, and the max |TE| will reach tens of microseconds, and it will be significantly affected by the increase in $N_{\mathrm{hop}}$.

Therefore, in a network with a switch hop count of less than four and BG traffic of less than 70 Mbps, our clock servo is sufficient to achieve 1 μs synchronization accuracy. Although the performance is not as good as the results using TC switches in ref. [19], it is significantly improved compared to using ordinary switches.

### 6.2. Comparison with Other Methods

We compare our method with the four design methods of the clock servo [27,28,31,33] and QIDACM [40]. The comparative fuzzy-PI servo includes [33] instead of [34] because hybrid IWCA-CS [34] seeks the optimal solution through continuous iterations, which has a large computational overhead. Our low-cost STM32 platform does not have enough computing capability. Moreover, some communication tasks and measurement and control tasks will be deployed in our nodes in the future, and the overhead of the clock synchronization task should be as small as possible. As introduced in Table 2, there are many QIDACMs, but they have respective limitations:

- Dedicated switches for PTPv2 are expensive.
- Sending probing packets causes major changes to PTP. Controlled packet departure has strict timing requirements. Their compatibility and applicability are not strong.
- PTP-LP has a high computational cost and is also not suitable for the STM32 platform.
- Optimal estimation algorithm requires an oscilloscope to provide input, which is not easy to implement in practical applications.

As a result, we focus on comparing the method in [40], which also uses the principle of detecting the minimum delay packet. Comparative performance metrics include time error, convergence time, and MCU utilization. When comparing the time error, the BG traffic is set from 10 to 70 Mbps. The BG traffic length is set to 1518 Bytes, and the switch hop count is set to one. Because the errors of some methods cannot converge within 1 μs, there is no BG traffic when comparing the convergence time, and the initial time offset is set to 1 ms. The MCU utilization is measured through the function vTaskGetRunTimeStats() provided by FreeRTOS.

The parameter settings of each method are organized in Table 6. For LF-PI [27], the time offset filter coefficient is set to 0.5, and the PI coefficients are set to 0.5 and 0.0625. The PI coefficients of the optimal PI [31] are both set to 1. The PI coefficients of the KF-PI [28] are also set to 1. The process noise covariance is set to 0.1 (μs)^2^, and the measurement noise covariance is calculated based on the one-way delay measured fifty times before starting the filter [48]. The core parameters of the fuzzy-PI [33] are consistent with those in this paper, except for the input physical domain. The upper bound of the absolute time offset is set to 500 μs, and that of the absolute offset derivative is set to 100 μs/s. The PTP synchronization period of the above four methods is set to 4 s, so the frequency correction period is also 4 s. Since ref. [40] only provides the time offset estimation method and does not include the correction method, we combine it with fuzzy-PI, and the fuzzy parameters are entirely consistent with those in this paper. Furthermore, it does not provide an estimation method for clock skew. For convenience of comparison, the clock skew directly uses the value of Equation (12) in this paper, with the opposite sign. The exponentially weighted moving average filter factor is set to 1.

**Figure 15:**
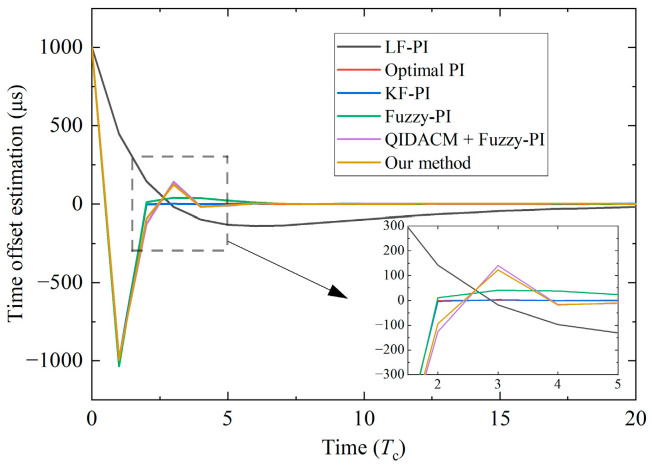
Convergence process of different methods (slave clock 2; initial time offset 1 ms; convergence condition |TE| ≤ 1 μs).

Table 7 summarizes the results of the six methods when BG traffic is 50 Mbps. Figure 15 is the convergence process of the time offset of slave clock 2. None of the first four methods can achieve 1 μs synchronization accuracy, and their mean error is approximately −10 μs. Optimal PI has the fastest convergence, requiring about four correction periods. However, its performance is the worst. The STD is about 100 μs, and the max |TE| reaches nearly 500 μs. Because the PI coefficients of this method are both 1, the loop bandwidth is the widest, the dynamic characteristic is the strongest, and the filter characteristic is the worst. Fuzzy-PI adds fuzzy logic compared to optimal PI, and the synchronization performance is improved. The STD is reduced to about 60 μs, and the max |TE| is reduced to about 300 μs. The convergence becomes slower and requires 11 correction periods. The synchronization performance will be significantly enhanced after adding a filter to the clock servo. The STD of LF-PI is about 35 μs, and the max |TE| is about 160 μs, but the convergence speed is the slowest, requiring about 40 correction periods. The reason is that the LF will introduce phase delay, and the system is in an overdamping state. Among these four methods, KF-PI has the best performance. The max |TE| is about 100 μs, and the convergence time is basically the same as the optimal PI.

QIDACM [40] and our method use the window filter based on oversampling, and the synchronization accuracy is improved to 1 μs. The performance of the two methods is relatively close, and the convergence time is seven correction periods. In comparison, the STD and max |TE| of our method are smaller. As the window length of the two methods is set to 32, in order to ensure that the frequency correction period remains unchanged, the PTP synchronization period is reduced to 125 ms, and the number of PTP packets increases significantly. Even so, the PTP traffic still accounts for less than 1% of the 100 Mbps bandwidth, which is completely acceptable for practical applications. Moreover, a large amount of PTP traffic also increases the processing burden of the node MCU, so we measure the MCU utilization of the six methods. When there is no BG traffic, the MCU utilization of the four design methods of the clock servo is less than 1%, while that of QIDACM [40] and our method is about 3%. Therefore, the burden of oversampling to the MCU is almost negligible, and the MCU still has enough time to handle other measurement and control tasks.

**Figure 16:**
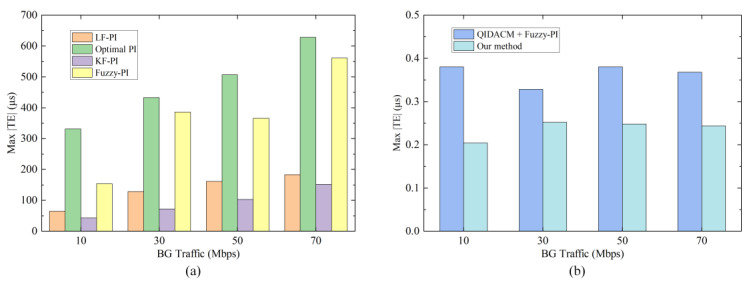
Comparison of the max |TE| of the six methods under different BG traffic. (**a**) Four design methods of the clock servo. (**b**) QIDACM [40] and our method.

We adjust the BG traffic to 10, 30, and 70 Mbps and conduct multiple experiments using the six methods above. In Figure 16a, we can see that the synchronization performance of the four design methods of the clock servo is easily affected by BG traffic. The greater the BG traffic, the worse the performance. The accuracy of these four methods ranges from tens of microseconds to hundreds of microseconds, and the performance ranking is KF-PI > LF-PI > Fuzzy-PI > Optimal PI. Figure 16b shows the comparison between QIDACM [40] and our method. Under different BG traffic, our method always performs better, and the max |TE| does not exceed 0.3 μs.

## 7. Conclusions

This paper designs a PTP clock servo for compensating QIDA, aiming at addressing the high cost and poor flexibility of the dedicated switches supporting TC. Its main algorithm consists of a minimum window filter with drift compensation and a fuzzy-PI controller. The minimum window filter is an oversampling nonlinear filter. Before drift compensation, the frequency offset within the window needs to be estimated. It is an optimal bidirectional estimation, effectively reducing the estimation error. The control system model of the clock servo is simplified by constructing a new input and output, and a method of determining the PI coefficients according to the damping ratio and natural frequency under the discrete system model is proposed. Adding fuzzy control can ensure fast convergence and high synchronization accuracy simultaneously. Finally, the performance of the clock servo is evaluated based on the low-cost experimental platform and the broadcast traffic model. Oversampling will only generate limited traffic, accounting for less than 1% of the 100 Mbps bandwidth. Furthermore, the burden of oversampling to the MCU is almost negligible, and the utilization is measured to be about 3%. When the switch hop count is less than four and the BG traffic is less than 70 Mbps, the max |TE| does not exceed 0.35 μs, and the convergence time is about half a minute. Compared with other existing clock servos, this synchronization accuracy is improved hundreds of times.

Future work can further conduct temperature experiments based on the existing hardware platform. In addition, the current BG traffic model is a fixed-length periodic UDP multicast packet, and we can study the performance of our clock servo under more complex traffic models.

## Figures and Tables

**Table 1:** Symbols and associated definitions.

Symbols	Definitions
x, $\tilde{x}$$,\;\hat{x}$	True value, measurement value, and estimation value of the time offset
$t_{1}$ $,\;t_{2}$ $,\;t_{3}$ $,\;t_{4}$	Four timestamps obtained through PTP packet exchanges
$t_{21}$ $,\;t_{43}$	Forward and backward timestamp differences
$t_{21}$ $,\;t_{43}$	Forward and backward timestamp difference vectors
$d_{\mathrm{ms}}$ $,\;d_{\mathrm{sm}}$	Forward and backward delays
$\kappa_{\mathrm{ms}}$ $,\;\kappa_{\mathrm{sm}}$	Forward and backward static delays
$\delta_{\mathrm{ms}}$ $,\;\delta_{\mathrm{sm}}$	Forward and backward queue delays
w	Measurement noise of the time offset
*n*	PTP synchronization process index
*V*	Constant value in the frequency compensation clock model
$u_{0}$ $,\;u_{\mathrm{com}}$	Initial value and compensation value of the addend
$f_{\mathrm{sys}}$ $,\;f_{0}$	System clock frequency and slave clock frequency
$T_{0}$	Slave clock period
*k*	Observation window index
${\hat{y}}_{21}$ $,\;{\hat{y}}_{43}$	Forward and backward frequency offset estimations
$\hat{y}$	Final frequency offset estimation
*N*	Observation window length
$\tau_{21f}$ $,\;\tau_{21b}$ $,\;\tau_{43f}$ $,\;\tau_{43b}$ $,\;\tau_{21}^{\prime }$ $,\;\tau_{43}^{\prime }$	Minimum timestamp differences
$m_{21f}$ $,\;m_{21b}$ $,\;m_{43f}$ $,\;m_{43b}$	Index of the minimum timestamp differences in the observation window
ψ{}	Operator of mathematical statistics
$T_{\mathrm{sync}}$ $,\;T_{c}$	PTP synchronization period and frequency correction period
$T_{M}$ $,\;T_{S}$	Master and slave clock time
e, ec	Time offset and its derivative
r	Remaining estimation error after window filtering
D(z) , G(z)	Transfer function of the PI controller and the clock
Φ(z)	Closed-loop Transfer function of the clock servo system
$\Phi_{s}\left(s\right)$	General closed-loop Transfer function of a second-order continuous system
$k_{c}$	Constant coefficient in the clock transfer function
$k_{p}$ $,\;k_{i}$	Proportional and integral coefficients
ξ	Damping ratio
$\omega_{n}$	Natural frequency
$B_{L}$	Equivalent noise bandwidth
$e_{f}$ $,\;e_{\mathrm{fc}}$ $,\;\omega_{f}$	Values of the input and output on the fuzzy domain
$k_{e}$ $,\;k_{\mathrm{ec}}$ $,\;k_{\omega }$	Scaling factors of the fuzzy controller
E, $E_{c}$$,\;\Omega_{d}$$,\;\Omega_{u}$	Boundary of the physical domain
$E_{f}$ $,\;E_{\mathrm{fc}}$ $,\;\Omega_{f}$	Boundary of the fuzzy domain
$W_{\mathrm{bg}}$	BG traffic
$L_{\mathrm{bg}}$	BG traffic packet length
$N_{\mathrm{hop}}$	Switch hop count
$T_{\mathrm{temp}}$	Ambient temperature

**Table 2:** Comparison of different QIDACMs.

QIDACMs	Accuracy	Hardware Support	Cost	Computational Complexity	Extra Packet Exchange	Compatibility	Applicability
PTPv2 [9]	1 μs	Yes, dedicated switch	High	Low	No	Poor	Good
Probing packets [36]	100 μs	No	Low	Low	Yes	Poor	Moderate
Controlled packet departure [37]	N/A	No	Low	Low	No	Poor	Poor
Filter [27,28]	1 ms	No	Low	Low	No	Good	Good
Packet selection algorithm [35,38,39,40,41]	1 μs	No	Low	Moderate	Yes	Good	Good
PTP-LP [42]	100 μs	No	Low	High	No	Good	Good
Optimal estimation algorithm [43]	1 μs	Yes, oscilloscope	Moderate	Moderate	No	Good	Poor

**Table 3:** Fuzzy control rules.

|*e*|/|*ec*|	NB	NS	ZO	PS	PB
**NB**	NB	NB	NB	NS	ZO
**NS**	NB	NS	NS	ZO	PS
**ZO**	NS	NS	ZO	PS	PS
**PS**	ZO	ZO	PS	PS	PB
**PB**	PS	PS	PS	PB	PB

**Table 4:** Different parameter types and values for clock synchronization.

Types	Parameters	Values
Algorithm parameters	Slave clock period $T_{0}$	7 ns
	$\mathrm{Synchronization}\;\mathrm{period}\;T_{\mathrm{sync}}$	125 ms
	Observation window length *N*	32
	PI coefficients $k_{p}$, $k_{i}$	Calculated by the fuzzy controller
External parameters	BG traffic $W_{\mathrm{bg}}$	From 0 to 100 Mbps
	BG traffic packet length $L_{\mathrm{bg}}$	512, 1024, and 1518 Bytes
	Switch hop count $N_{\mathrm{hop}}$	From 1 to 5
	Ambient temperature $T_{\mathrm{temp}}$	Room Temperature

**Table 5:** Results of the PI coefficients experiment.

ξ.	$\omega_{n}$ (rad/s)	Time Error (μs)									$\mathrm{Convergence}\;\mathrm{Time}\;(T_{c}$)		
		Slave Clock 1			Slave Clock 2			Slave Clock 3			Slave Clock 1	Slave Clock 2	Slave Clock 3
		Mean	STD	Max |TE|	Mean	STD	Max |TE|	Mean	STD	Max |TE|			
0.707	0.05	0.011	0.129	0.476	−0.087	0.188	0.656	−0.031	0.208	0.664	46	46	46
	0.1	0.010	0.065	0.228	−0.051	0.087	0.292	−0.022	0.087	0.304	23	23	23
	0.2	−0.016	0.041	0.168	−0.050	0.051	0.228	−0.036	0.055	0.244	12	12	12
	0.3	−0.007	0.038	0.188	−0.034	0.040	0.208	−0.022	0.055	0.260	10	10	10
	0.4	0.017	0.049	0.292	−0.020	0.047	0.228	−0.015	0.058	0.288	8	8	7
	0.5	0.006	0.053	0.300	−0.012	0.052	0.268	−0.003	0.061	0.248	8	8	8
	0.75	−0.033	0.070	0.460	−0.018	0.062	0.352	−0.035	0.071	0.344	7	7	7
	1	0.005	0.057	0.360	−0.006	0.063	0.532	0.008	0.061	0.304	7	7	8
	5	−0.024	0.064	0.432	−0.018	0.054	0.364	−0.018	0.059	0.316	7	7	7
Fuzzy-PI		0.005	0.039	0.168	−0.006	0.046	0.248	−0.005	0.057	0.196	8	7	8

**Table 6:** Parameter settings of different clock synchronization methods.

Methods	Parameters	Values
LF-PI [27]	Time offset filter coefficient	0.5
	PI coefficients	0.5 and 0.0625
Optimal PI [31]	PI coefficients	1
KF-PI [28]	Process noise covariance	0.1 (μs)^2^
	Measurement noise covariance	Calculated based on the one-way delay measured fifty times before starting the filter
	PI coefficients	1
Fuzzy-PI [33]	Upper bound of the absolute time offset	500 μs
	Upper bound of the absolute offset derivative	100 μs/s
QIDACM [40]	Clock skew	Value of Equation (12) in this paper, with the opposite sign
	Exponentially weighted moving average filter factor	1
All	Synchronization period	4 s

**Table 7:** Comparison of our method with other clock synchronization methods: time error and convergence time.

Methods	Time Error (μs)									$\mathrm{Convergence}\;\mathrm{Time}\;(T_{c})$		
	Slave Clock 1			Slave Clock 2			Slave Clock 3			Slave Clock 1	Slave Clock 2	Slave Clock 3
	Mean	STD	Max |TE|	Mean	STD	Max |TE|	Mean	STD	Max |TE|			
LF-PI [27]	−10.8	34.2	157	−9.73	33.1	134	−10.5	32.5	161	40	38	38
Optimal PI [31]	−9.03	98.3	507	−11.0	94.5	376	−11.0	106	465	4	4	3
KF-PI [28]	−12.0	44.5	100	−10.0	55.1	103	−10.3	23.0	55.3	4	4	4
Fuzzy-PI [33]	−9.36	54.7	366	−9.37	53.1	264	−10.2	55.4	306	11	11	11
QIDACM [40] + Fuzzy-PI	0.009	0.051	0.380	−0.001	0.056	0.260	0.010	0.069	0.364	7	7	7
Our method	0.005	0.039	0.168	−0.006	0.046	0.248	−0.005	0.057	0.196	7	7	7

## Data Availability

The data presented in this study are available from the corresponding author upon request. The data are not publicly available due to privacy restrictions.
